# Risk of drowning in Dez River as the most dangerous river of Iran: a case study 

**Published:** 2022-12

**Authors:** Hamidreza Aghababaeian, Abbas Ostadtaghizadeh, Laden Araghi Ahvazi, Fateme Yazdi, Mohammad Maniey, Fereidoon Jafarian

**Affiliations:** ^*a*^ Center for Climate Change and Health Research (CCCHR), Dezful University of Medical Sciences, Dezful, Iran.; ^*b*^ Department of Medical Emergencies, Dezful University of Medical Sciences, Dezful, Iran.; ^*c*^ Department of Health in Emergencies and Disasters, School of Public Health, Tehran University of Medical Sciences, Tehran, Iran.; ^*d*^ Center for Climate Change and Health Research (CCCHR), Institute for Environmental Research (IER), Tehran University of Medical Sciences, Tehran, Iran.; ^*e*^ Dezful University of Medical Sciences, Dezful, Iran.

## Abstract

**Background:**

The evidence shows that the Dez River in Dezful city of Khuzestan province is one of the most dangerous places in Iran where drowning usually occurs. This study was conducted with the aim of examining the risk of drowning and introducing the Dez River as one of the most important drowning sites in the country, which requires preventive measures.

**Methods:**

This case study used the official data obtained from the Forensic Medicine Organization, Red Crescent, Emergency and Dezful Municipality, literature review, evidence, and information from field observation related to the risk of drowning in the Dez River in 2022. To complete the information, observation and field survey were also used.

**Results:**

Between 2012 and 2021, an average of 1042 drownings were reported in Iran each year. The highest number of drowning victims in the country in the last decade was in Khuzestan province, with an average of 140 deaths per year. Official forensic information shows that the highest death toll in Khuzestan is linked to drowning in the Dez River. Moreover, between 1998 and 2019, more than 573 people drowned in this river (an average of 28 people per year). Despite the constant annual trend of fatalities in the river, field observations have indicated that some parts of Dez still lack the required facilities and rescue equipment for drowning prevention.

**Conclusions:**

Drowning in the Dez River has made the city of Dezful one of the most important cities in the world considering the drowning issue. Therefore, the Dez River should be prioritized as one of the most potentially high-risk places for drowning reduction in Iran. This problem requires urgent scientific measures to reduce drowning and its related injuries.

**Keywords:**

Drowning, Emergencies, Dez river, Risk, Dezful

